# The differential impacts of non-locally acquired infections and treatment interventions on heterosexual HIV transmission in Hong Kong

**DOI:** 10.1371/journal.pone.0237433

**Published:** 2020-08-13

**Authors:** Ngai Sze Wong, Man Po Lee, Ka Hing Wong, Owen T. Y. Tsang, Shui Shan Lee

**Affiliations:** 1 Stanley Ho Centre for Emerging Infectious Diseases, The Chinese University of Hong Kong, Shatin, Hong Kong, People’s Republic of China; 2 Institute for Global Health & Infectious Diseases, University of North Carolina at Chapel Hill, Chapel Hill, NC, United States of America; 3 University of North Carolina Project-China, Guangzhou, Guangdong, China; 4 Department of Medicine, Queen Elizabeth Hospital, Kowloon, Hong Kong, People’s Republic of China; 5 Special Preventive Programme, Department of Health, Hong Kong Special Administrative Region Government, Hong Kong, People’s Republic of China; 6 Department of Medicine and Geriatrics, Princess Margaret Hospital, Lai King, Kowloon, Hong Kong, People’s Republic of China; University of Sheffield, UNITED KINGDOM

## Abstract

**Introduction:**

Heterosexual infections have contributed to a high proportion of the HIV burden in Asia and Eastern Europe. Human mobility and non-local infections are important features in some cities/countries. An understanding of the determinants of the sustained growth of the heterosexual HIV epidemics would enable the potential impacts of treatment-based interventions to be assessed.

**Methods:**

We developed a compartmental model for heterosexual HIV transmissions, parameterized by clinical and surveillance data (1984–2014) in Hong Kong. HIV sequence data were included for examining genetic linkages and clustering pattern. We performed sensitivity analyses to evaluate effects of high-risk sexual partnership and proportions of non-locally acquired infections. Four hypothetical interventions (a) immediate treatment, (b) enhancement of retention in care, (c) HIV testing campaigns, and (d) test-and-treat strategy, were examined.

**Results:**

Data of 2174 patients (723 female and 1451 male) diagnosed with HIV between 1984 and 2012 in Hong Kong were collected for model parameterization. Among 1229 sequences of non-MSM (men who have sex with men) patients, 70% were isolates and 17% were either dyads or triads. In base-case scenario, the total estimated number of new infections in 2012–2023 would be 672 for male and 452 for female. Following 100% retention in care intervention, the total proportion of averted new infections in 2012–2023 would be 7% for male and 10% for female. HIV testing campaign in 2012 and 2017 followed by 100% immediate treatment strategy would avert 5% and 9% of male and female new infections, respectively. In the epidemic model, an increase of high-risk sexual partnership from 6% to 9% would increase the epidemic growth (annual number of newly diagnosed and newly infected cases) by about 10%. If no non-locally acquired infection occurred as from 2012, the epidemic growth would slump. To control the heterosexual epidemic, periodic HIV testing at 5-year intervals with immediate treatment would avert 5–13% of annual new infections in 2013–2023.

**Conclusions:**

Enhanced HIV testing with immediate treatment is most effective in controlling the heterosexual epidemic, the impacts of which might however be attenuated by any increase of non-locally acquired infection, assuming little variations of high risk partnership over time.

## Introduction

Worldwide, sexual transmission accounts for a majority of prevailing HIV infections. In the past decade, HIV transmission in men who have sex with men (MSM) has escalated globally, an observation also seen in Asian countries. [1, 2] In contrast, delineation of epidemiological patterns in heterosexual infection has so far received relatively less attention, [3–6] especially outside sub-Sahara Africa. In Asia and Eastern Europe, heterosexual infections have contributed significantly to the overall HIV viral burdens at population levels. [7] In 2017, for example, heterosexual transmission accounted for 68% of all reported new HIV diagnoses in Eastern Europe. [8] In the development of intervention programmes, key populations including MSM, people who inject drugs (PWID) and female sex workers (FSW) have often been the main focus of HIV prevention programme, while heterosexually infected persons are neglected. Despite much lower risk of HIV transmission, the large population size could mean a high number of HIV infections and the challenge of effective prevention program implementation. When transmission of HIV in the key populations could explain the emergence of concentrated HIV epidemics, it could not well explain the sustained transmissions of the virus in the general population.

Partner concurrency could be a driver of the generalized heterosexual HIV epidemics in Africa, though such proposition has continued to be hotly debated. [9–11] The predicted impacts of interventions targeting concurrency were however moderate. [9] The existence of small networks, impacted by concurrency, has been examined through stochastic modelling to understand the sustained transmission of the virus in the community. [12] To explain the sustained heterosexual transmission, non-locally acquired infections were suggested to be another key parameter. In Europe, migrants accounted for 41% of new HIV diagnoses in 2017. The declining trend of reported heterosexual cases was affected by the falling number of heterosexual migrant cases originating from generalized HIV epidemic countries. [8] In Australia, 35% of heterosexual HIV diagnoses in 2002–2011 were attributable to non-local exposure. [13] Some HIV epidemic modelling studies have also focussed the influence of migration on local epidemic growth, the impact of which varied by local context. [14–16]

In Hong Kong, an international city in Asia, the heterosexual HIV epidemic grew rapidly in the 1980s-1990s but became stabilized from 2000 onwards. Heterosexual infections have accounted for some two-fifths of all reported cases despite the low overall HIV prevalence in the population, [17] a scenario shared by other low prevalence countries in Asia. Following expanding coverage of antiretroviral therapy (ART) for HIV-infected individuals in care, the impacts of treatment-as-prevention could play a role in shaping future epidemiology. To explain the transmission pattern of heterosexual HIV infections, a compartmental model was designed to assess the potential impacts of interventions centring on the promotion and enhancement of ART, and non-locally acquired infections.

## Methods

### Model structure

A deterministic compartmental model was constructed in R3.5.1, composing of a heterosexual male and a heterosexual female sub-model, with interactions between but not within the two. Bisexual and transgender sub-models were not separately constructed but subsumed under MSM, the epidemiology of whom was not modelled in this study. Each sub-model was structured by compartments of susceptible (sexually active adults aged 15–64 for female and aged 15–84 for male) and infected individuals progressing through the following disease stages–acute infection, chronic infection categorized by CD4 levels of ≤200/μL, 201-350/μL, 351-500/μL, >500/μL, and AIDS–and along the cascade of HIV care (undiagnosed, diagnosed, treatment initiated and lost to follow-up) (**S1 Appendix**). The model was an open system with population inflow (net susceptible population growth and non-locally acquired infections) and outflow (death). The epidemic growth was simulated from 1983 to 2011, and projected from 2012 to 2023.

### Data source and model parameters

Anonymous longitudinal clinical data in 1985–2014 of HIV-infected heterosexuals aged 18 or above at diagnosis were retrieved from an HIV cohort database built with data accessed retrospectively from all three HIV specialist clinics in the public service in Hong Kong. [18] In addition, HIV-1 genotype resistance testing sequences in 1994–2012 of HIV-infected non-MSM patients were accessed. Descriptive statistics were performed to describe the demographics, baseline clinical status and HIV treatment status of these individuals.

The collected data were used to estimate the value of most of the model parameters, including diagnosis rate, pre-treatment disease progression rate, treatment initiation rate, loss to follow-up rate, viral load suppression (SVL, ≤500 copies/mL) and rebound (>500/mL) rate (description on the derivation of parameter values from clinical data in **S1 Appendix**). Annual number of reported new diagnoses and proportion of suspected non-local HIV infection were retrieved from surveillance epidemiological datasets. Parameters from overseas clinical trials, such as transmission risk, were referenced from literature.

Approvals of the Joint Chinese University of Hong Kong–New Territories East Cluster Clinical Research Ethics Committee (CREC), Kowloon West Cluster Research Ethics Committee, and Kowloon East Cluster Research Ethics Committee were obtained. Data access approval was granted by Department of Health, Hong Kong Special Administrative Region Government in compliance with the Personal Data (Privacy) Ordinance. Informed consent was waived as the collected data were anonymised and had been collected retrospectively. This study was conducted in compliance with Declaration of Helsinki.

To describe the clustering pattern of cases, collected HIV-1 sequences (protease and partial reverse transcriptase of pol gene) were aligned by MUSCLE and analysed by neighbour-joining tree in MEGA6 to identify clusters with bootstrap value ≥90, 1000 times simulation. A total of 1229 sequences of patients were analysed (976 for heterosexual contacts, 18 for contaminated blood transfusion contacts, 204 for injection drug use, and 31 for undefined transmission mode). Among them, 142 sequences (12%) were genetically connected with 1 other sequence, forming 71 dyads, and another 60 sequences (5%) were connected with 2 other sequences in 20 small triads, 164 sequences (13%) were connected with at least 3 other sequences in 23 clusters (**S1 Appendix, p.9**), and the rest (70%) were isolated sequences. Different from HIV-infected MSM who formed a number of clusters, [19] most of the sequences were isolates, dyads or triads for HIV-infected heterosexuals. In this model, there were no further delineation of population in heterosexual male and female submodels. Instead, sexual partnership was ranked in our model. A susceptible individual was assumed to be at 1) high risk of HIV transmission if he/she and his/her sex partners were in concurrent partnership; 2) medium risk if one was in serial monogamy while the other in concurrent partnership; 3) low risk if both partners were in serial monogamy. We further assumed that the risk decreased by one level after HIV diagnosis, as a result of ART and/or reduction of behavioural risk. [20] Estimation of the force of infection is described in the **S1 Appendix**.

We fitted model predictions over time to the reported prevalent number of HIV-infected heterosexual male and female in 1984–2012 in Hong Kong under negative log likelihood in R (stats4 package) using male function. In this calibration process, [21] we simultaneously varied the duration of partnership for male and female at medium risk, duration and proportion of partnership at low risk within data ranges suggested in the previous studies to find the best fit value. Modelling results were validated with number of reported prevalent cases in 2013 and annual number of newly diagnosed cases. [22]

We defined non-locally acquired infections as HIV transmissions occurring either outside Hong Kong, or from a non-local HIV positive individual within the city. It was categorized by gender and ethnicity (Chinese and non-Chinese) in model parameters. A Chinese person with non-locally acquired HIV infection was assumed to be physically present in Hong Kong during acute infection, on the premise that he/she was either infected during short travel overseas or had contracted the virus from non-local persons within the territory. Non-locally acquired HIV infection in a non-Chinese was however added to the compartment of patients diagnosed with CD4>500/μL, assuming that they were infected beyond the period of acute infection before entering Hong Kong. Annual surveillance reports [17] published by the Department of Health were consulted to derive parameter values.

### Scenarios constructions

In base-case scenario, we simulated the HIV epidemic in 1983–2010, parameterized by longitudinal clinical data and literature. The epidemic growth for heterosexuals in the medium term (2012–2023) was then projected with treatment initiation criteria and diagnosis rate of 2011. In response to the recommended test-and-treat strategy for controlling the HIV epidemic, [23–25] four scenarios of treatment-based interventions were designed for introduction from 2012, while keeping other conditions unchanged. These scenarios were A) rate of retention in care before and on ART at 50% and 100% from 2012; B) immediate treatment for all diagnosed cases from 2012; C) HIV testing campaigns in 2012 and 2017 (assuming 100% diagnosis rate in the respective year); and D) test-and-treat–HIV testing campaign in 2012 and 2017 with immediate treatment from 2012.

### Sensitivity analyses

To examine the impact of uncertainty of key parameters on model simulation results, sensitivity analyses were performed for the influences of varying the risk ranking ratios of sexual partnership, and proportion of non-locally acquired infections, with the starting point of 1) 1983 and 2) 2012 respectively. Five combinations of partnership types were simulated in the sensitivity analyses by varying the ratios of high:medium:low risk partnership: A) 6%:38%:56% (base-case); B) 3%:38%:59%; C) 3%:41%:56%; D) 9%:35%:56%; and E) 9%:38%:53%. Sensitivity analyses for zero, twice and thrice the annual number of non-locally acquired infections by gender were performed. Other model parameters were kept unchanged and no intervention were introduced in these analyses. Separately, sensitivity analyses for non-locally acquired infections were performed from 2012 with intervention scenarios.

## Results

### Characteristics of heterosexually acquired HIV infections in Hong Kong

Between 1984 and 2012, 2174 (48%) of 4508 patients aged 18 or above at diagnosis in the cohort database had contracted HIV through heterosexual contacts. Of these, 1844 (85%) were diagnosed in or after 1997, when ART became regularly offered to patients under a CD4-guided approach. The male-to-female ratio of these patients was 2:1. CRF01_AE was the commonest HIV subtype, accounting for 66% of patients whose virus had been genotyped. The patients’ characteristics were compared by gender using univariate analyses (odds ratio) and Mann-Whitney *U* test (*U*-test). Females were more likely to be non-Chinese and were younger at diagnosis compared to males (33 year-old vs 40 year-old, p<0.001). (**Table 1**) Overall, males were at a poorer clinical state at diagnosis with a lower median CD4 count (116/μL vs 205/μL, p<0.001) and higher viral load (median log_10_ 4.9/mL vs log_10_ 4.6/mL, p<0.01). A higher proportion of male had progressed to AIDS at HIV diagnosis, or that the diagnosis was made within three months of AIDS (defined as late diagnosis in this study).

**Table 1:** General characteristics of heterosexually acquired HIV infections diagnosed between 1984 and 2014 in Hong Kong (n = 2174).

	Female			Male		
	n	%	N	n	%	N
	median	IQR		median	IQR	
**Demographics**						
Age at diagnosis*	33	28–41	723	40	33–49	1451
age >64 at diagnosis*	11	2%	723	102	7%	1451
Ethnicity						
Chinese*	349	48%	722	1157	80%	1450
Caucasian*	12	2%	722	69	5%	1450
Asian non-Chinese						
other ethnicity*	361	50%	722	224	15%	1450
**Baseline clinical status**						
HIV subtype						
CRF01_AE	265	66%	402	456	60%	755
B*	36	9%	402	169	22%	755
Others	101	25%	402	130	17%	755
^#^Late HIV diagnosis*	185	26%	723	541	37%	1451
AIDS at HIV diagnosis*	236	33%	723	712	49%	1451
Baseline CD4*	205	37–370	637	116	25–347	1266
≤200/μL*	316	50%	637	747	59%	1266
Baseline log viral load*	4.6	3.9–5.2	589	4.9	4.3–5.4	1097
>log_10_ 5/mL*	189	32%	589	493	45%	1097
**HIV Treatment**						
on ART	562	78%	723	1135	78%	1451
Months from diagnosis to ART	5	1–36	562	4	1–25	1135
Months from treatment initiation to SVL	3	2–6	514	3	3–6	1032
Regimen						
NNRTI-based	238	45%	534	501	46%	1091
PI-based	372	70%	534	760	70%	1091
SVL at the latest consultation	498	77%	648	971	79%	1230

*statistical significance as reflected by p<0.05, for comparison of odds ratio or Mann-Whitney U test

^#^Late diagnosis = HIV diagnosis made within 3 months of a diagnosis of AIDS

ART = antiretroviral therapy; NNRTI = non-nucleoside reverse transcriptase inhibitor; PI = protease inhibitor

SVL = suppressed viral load

At the latest assessment, 78% of the patients have been or were on ART. There was no gender difference as regards treatment status and responses. The median interval from diagnosis to ART initiation, and from treatment initiation to SVL, was four months and three months respectively. Overall, some 78% (1469/1878) had achieved SVL.

### Base-case model and its projection to 2023

**Fig 1:**
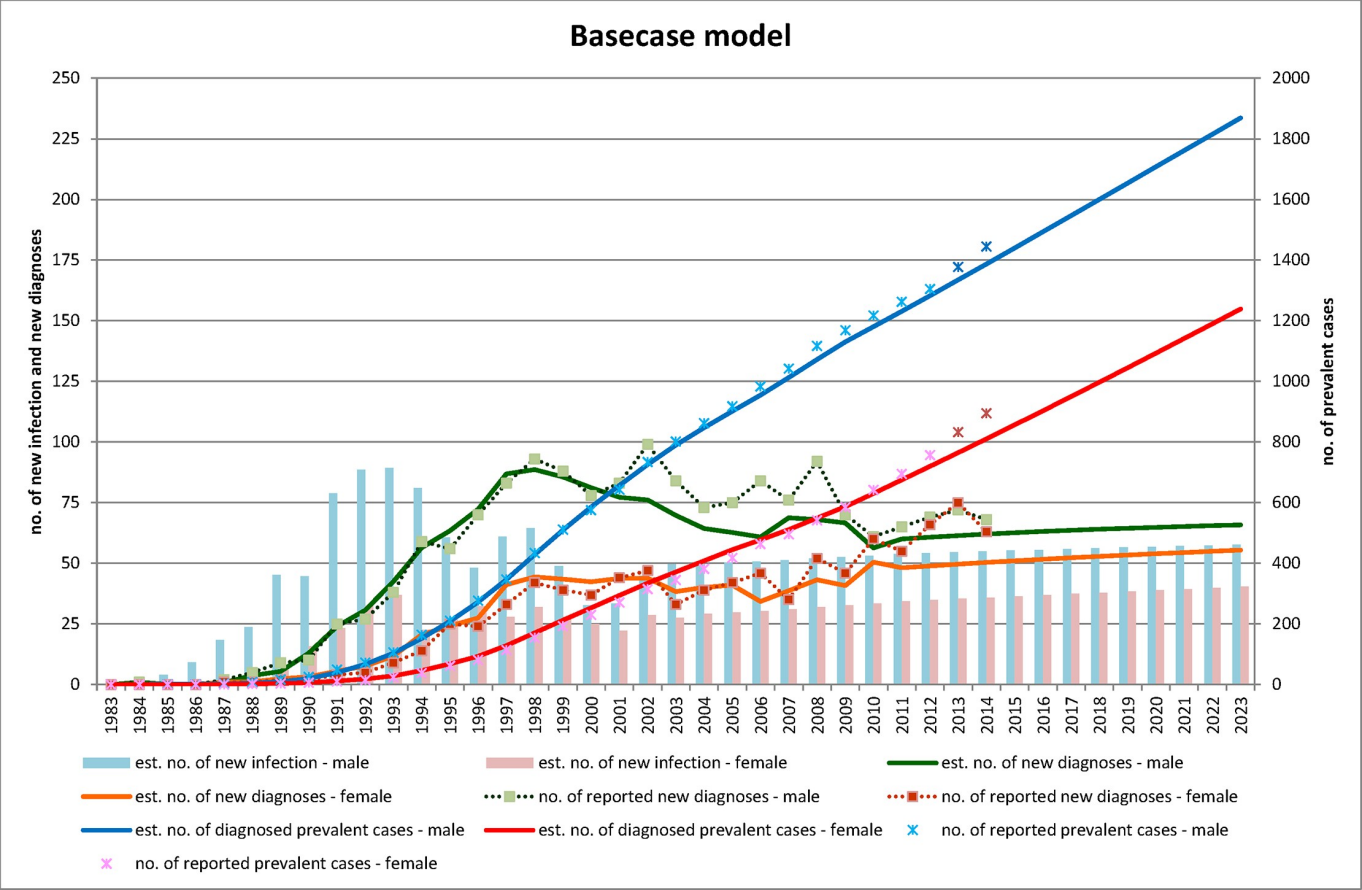
Projection of epidemic growth of heterosexual HIV infection in base-case model (1983–2023).

The model estimates of annual number of new diagnoses and prevalent diagnosed cases were similar to the reported numbers for the respective years in 1984–2012, except for a modest underestimation of the newly diagnosed male in 2002–2008, and slight underestimation of the prevalent diagnosed cases in 2013.(**Fig 1**) In base-case scenario, HIV infections (including undiagnosed individuals) would increase by 37% (from 2555 in 2014 to 3503 in 2023), with male-to-female ratio falling slightly from 1.74:1 to 1.54:1. The total number of new infections would be 672 for male and 452 for female in 2012–2023. The profile of HIV infections, treatment uptake, retention in care and viral load suppression by 2023 is shown in **Table 2**.

**Table 2:** Projection of heterosexually acquired HIV infections to 2023 in the base-case model.

	Total			Male			Female		
	2014	2023	% change	2014	2023	% change	2014	2023	% change
Undiagnosed	431	472	10%	276	294	7%	155	178	15%
New infections	91	98	8%	55	58	5%	36	40	11%
% local new infections	50%	53%	4%	38%	41%	3%	66%	70%	4%
Diagnosed prevalent cases	2198	3108	41%	1387	1869	35%	811	1239	53%
Newly diagnosed	112	121	8%	62	66	6%	50	55	10%
Diagnosed but not retained	405	446	10%	255	265	4%	150	181	21%
Diagnosed in care without treatment	186	196	5%	99	102	3%	87	94	8%
Treatment initiated but lost to followup	130	208	60%	85	128	51%	45	80	78%
On treatment	1404	2181	55%	909	1334	47%	495	847	71%
% with SVL in the community*	58%	65%	7%	59%	66%	7%	54%	63%	9%
% SVL in the population^#^	49%	57%	8%	49%	57%	8%	45%	55%	10%

SVL—suppressed viral load

*% with SVL in the community is calculated by dividing the number of individuals with SVL by the total number of diagnosed individuals in the year

^#^% with SVL in the population is calculated by dividing the number of individuals with SVL by the total number of undiagnosed and diagnosed individuals in the year

### Scenarios of interventions and parameter changes from 2012

**Fig 2:**
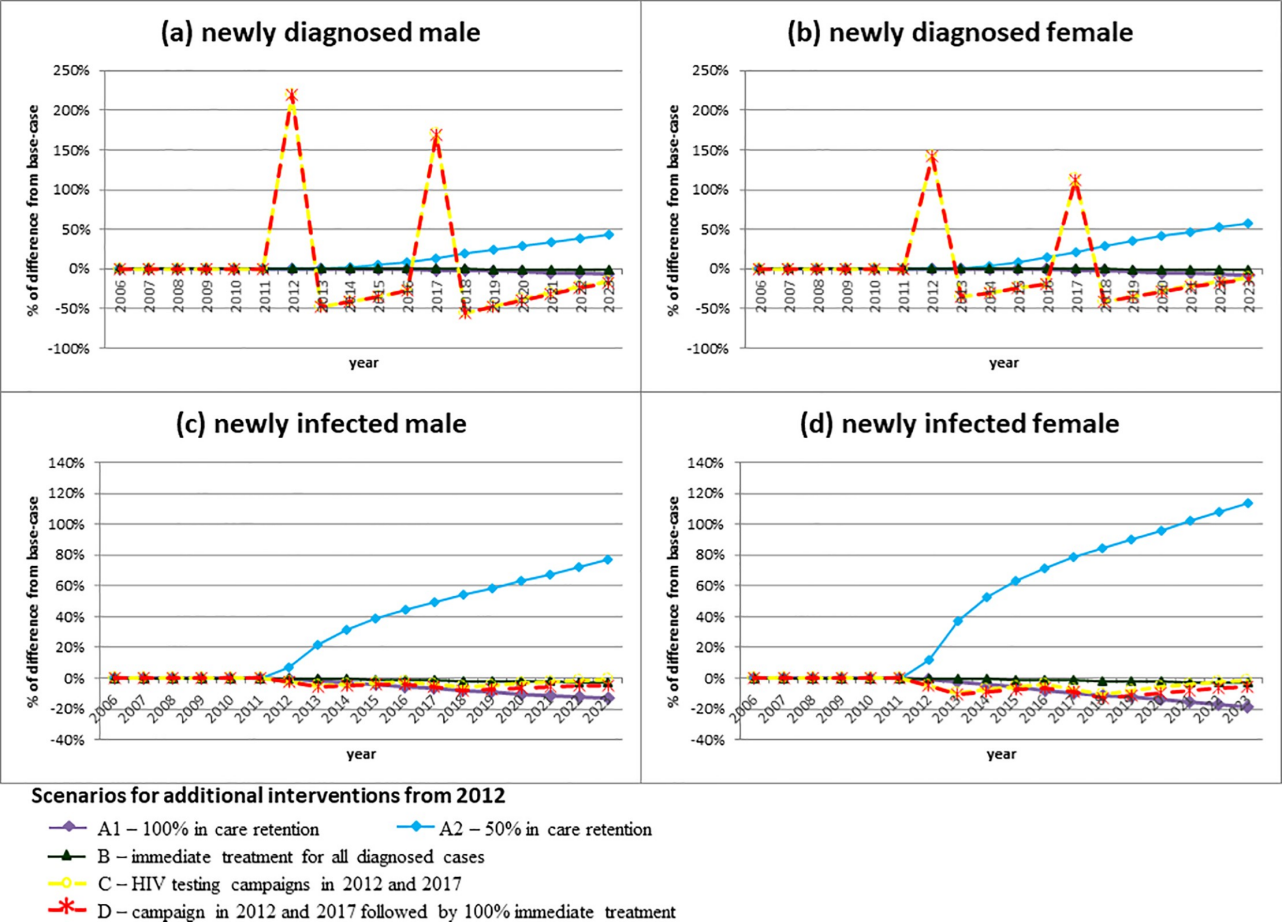
Proportion of change in (a-b) the number of newly diagnosed and (c-d) newly infected cased above base-case resulting from the five intervention scenarios introduced from 2012.

In the simulation, full retention in care *per se* (Scenario A1) resulted in lower epidemic growth than implementing immediate treatment alone (Scenario B) (**Fig 2, Table 3A and 3B**). If retention in care dropped to 50% (Scenario A2), the proportion of SVL would be halved by 2023. The annual number of new infections would increase sharply from 2012, and the total number of male and female new infections in 2012–2023 would be 330 (49%) and 347 (77%) more than base-case, respectively. Immediate treatment alone (Scenario B) would lead to minimal change of new infections above base-case. Scenario C (HIV testing campaign) led to the 3% male and 6% female new infections averted in 2012–2023. Moderate additive effects could result when HIV testing campaigns were combined with immediate treatment (Scenario D), as shown by an additional 4% reduction of estimated total new infections by 2023.

**Table 3:** Changes in heterosexually acquired infection following interventions introduced in 2012, as compared to base-case by 2023. A1–100% retention in care; A2–50% retention in care; B–immediate treatment for all diagnosed cases; C–HIV testing campaigns in 2012 and 2017; D–campaign in 2012 and 2017 followed by 100% immediate treatment.

**3(a) Male**						
	**Base-case**	**A1**	**A2**	**B**	**C**	**D**
	(Projected value)	Proportion change compared to base-case				
Undiagnosed (2023)	(295)	-10%	61%	-2%	-9%	-12%
New infections (2012–2023)	(672)	-7%	49%	-1%	-3%	-5%
% of local new infections (2012–2023)	(36%)	-5%	21%	-1%	-2%	-3%
Diagnosed prevalent cases	(1869)	-1%	7%	0%	1%	1%
Newly diagnosed (2012–2023)	(764)	-2%	18%	-1%	1%	0%
Diagnosed but not retained (2023)	(266)	-30%	118%	-12%	8%	-10%
Diagnosed in care without treatment (2023)	(102)	7%	-12%	-38%	-17%	-53%
Treatment initiated but lost to followup (2023)	(132)	-87%	667%	5%	2%	7%
On treatment (2023)	(1369)	12%	-76%	5%	1%	6%
% with SVL in the community* (2023)	(67%)	10%	-58%	3%	1%	5%
% with SVL in the population^#^ (2023)	(57%)	9%	-50%	3%	2%	6%
**3(b) Female**						
	**Base-case**	**A1**	**A2**	**B**	**C**	**D**
	(Projected value)	Proportion change compared to base-case				
Undiagnosed (2023)	(180)	-14%	96%	-2%	-9%	-13%
New infections (2012–2023)	(452)	-10%	77%	-2%	-6%	-9%
% of local new infection (2012–2023)	(63%)	-4%	16%	-1%	-2%	-3%
Diagnosed prevalent cases (2023)	(1239)	-2%	13%	0%	0%	-1%
Newly diagnosed (2012–2023)	(629)	-3%	27%	0%	-1%	-2%
Diagnosed but not retained (2023)	(183)	-42%	156%	-19%	5%	-17%
Diagnosed in care without treatment (2023)	(94)	7%	-7%	-45%	-12%	-54%
Treatment initiated but lost to followup (2023)	(83)	-90%	651%	8%	1%	10%
On treatment (2023)	(878)	14%	-62%	8%	-1%	8%
% with SVL in the community* (2023)	(63%)	11%	-55%	5%	1%	6%
% with SVL in the population^#^ (2023)	(55%)	10%	-48%	5%	2%	7%

SVL—suppressed viral load

*% with SVL in the community is calculated by dividing the number of individuals with SVL divided by the total number of diagnosed individuals in the year

^#^% with SVL in the population is calculated by dividing the number of individuals with SVL by the total number of undiagnosed and diagnosed individuals in the year

**Fig 3:**
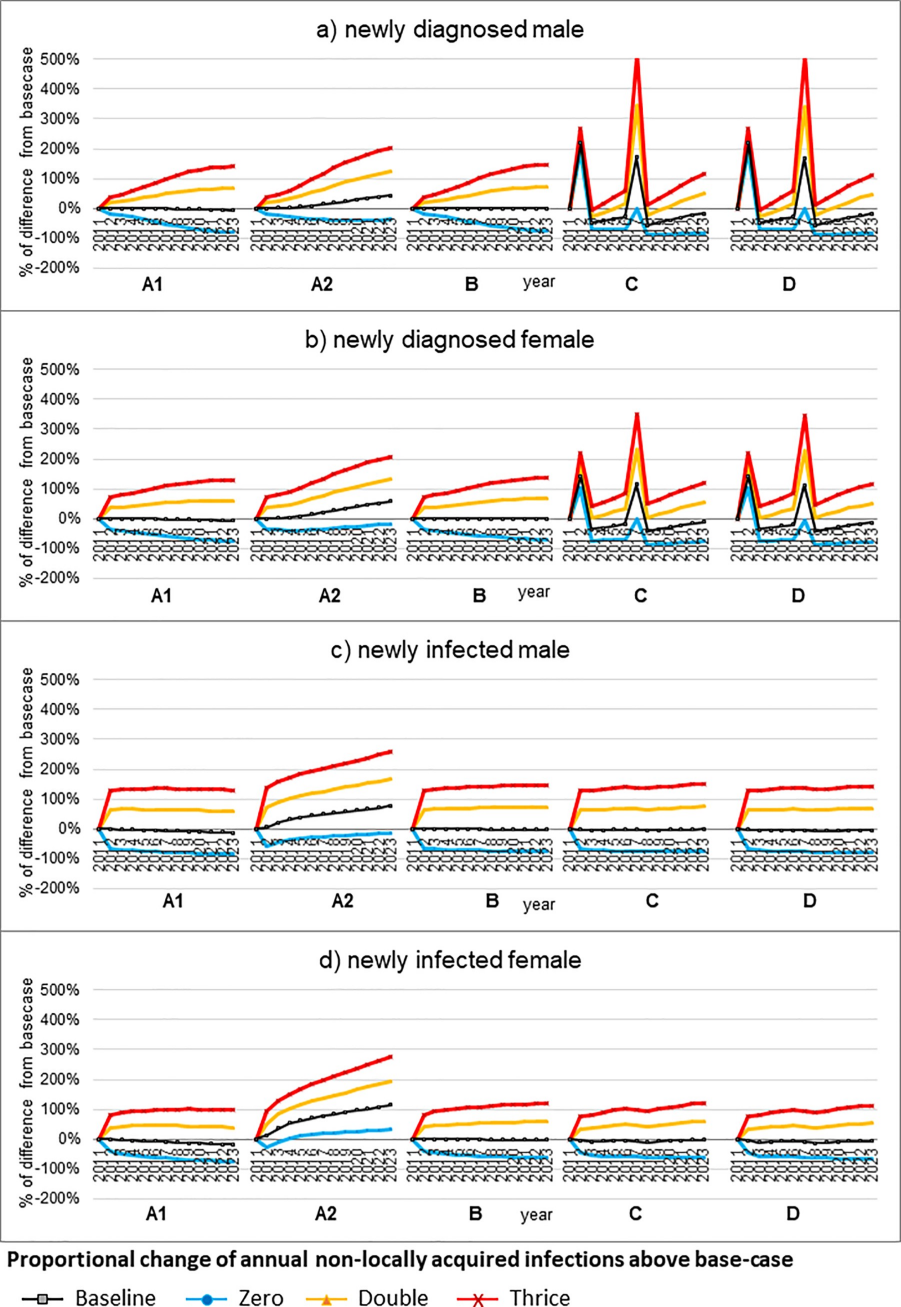
Sensitivity analyses with varying the proportion of non-locally acquired infection under five intervention scenarios (A1, A2, B, C, D) introduced from 2012. Scenario A1–100% retention in care; Scenario A2–50% retention in care; Scenario B–immediate treatment for all diagnosed cases; Scenario C–HIV testing campaigns in 2012 and 2017; Scenario D–campaign in 2012 and 2017 followed by 100% immediate treatment.

As shown in the sensitivity analyses, the effect of interventions was tested by changing the annual number of non-locally acquired infections. In Scenario D, the estimated annual number of new male diagnoses would increase from 53 (-14% above base-case) in 2014 to 96 (46% above base-case) in 2023 when the number of non-locally acquired infections doubled (**Fig 3**). Similarly, the male and female new infections above base-case would become 40 (69%) and 22 (53%) more, respectively, if the number of non-locally acquired infections doubled.

Without interventions, when no non-locally acquired infection was added to the model from 2012, the annual number of newly diagnosed and prevalent cases dropped linearly (**S1 Appendix, p.11**). The number of new diagnoses in 2023 would be 89 less (73% lower) than base-case, while the number of diagnosed prevalent cases would be 698 less (22% lower). Instead of a linear decline in annual new diagnoses, the number of new infections would be 37 and 16 less for male (68% lower) and female (46%) than base-case respectively in 2013, and this proportion of reduction would almost remain unchanged afterwards.

### Results of sensitivity analyses by parameter changes from 1983

From the sensitivity analyses with 1983 as the starting year, a higher proportion of high risk partnership (Scenario D and E in **S1 Appendix, p.12**) would drive the epidemic growth. Specifically, a 50% increase of high risk partnership (say, from 6% to 9%) generated about 10% increase in the annual number of newly diagnosed, newly infected and diagnosed prevalence cases. The resultant annual increase of new infections would be at least 5% higher in female compared to males since 2003. The impact of changing the proportional distribution of risk ranking of sexual partnership since 2012 on the epidemic growth (**S1 Appendix, p.13**) could be inferred from the changes of parameter values from 1983 in the sensitivity analyses.

In the absence of non-locally acquired infections from 1983, there would have been zero epidemic growth (results not shown). When the annual number of non-locally acquired infections doubled, the number of newly infected, newly diagnosed and diagnosed prevalent cases would likewise double. The epidemic growth differed when the proportion of male or female non-locally acquired infection was altered separately (**S1 Appendix, p.14**). By setting non-locally acquired male infections to zero, less than 400 newly diagnosed and newly infected male resulted in 1983–2023 (at least 70% lower than base-case) while there would be 896 newly diagnosed (39% lower) and 513 newly infected female (58% lower). Conversely, setting non-local female infections to zero would simulate 473 (61% lower) and 585 (42% lower) new female diagnoses and infections than base-case, while male epidemic growth became just 19% and 23% lower, respectively. Similarly, an increase of non-locally acquired male infections resulted in substantial growth of both the male and female epidemics, while an increase of non-locally acquired female infections impacted the female epidemic but caused minimal changes to the male epidemic.

## Discussion

In analysing the heterosexual HIV epidemic, our deterministic compartmental model has incorporated the inflow of non-locally acquired infection, configured by the mixing of males and females with different levels of sex partnership. Using concurrent sex partnership as a key parameter, the model provides a new explanation for interpreting the epidemiology of heterosexual HIV infections in Hong Kong. Concurrent partnership aside, non-locally acquired infection was of notable importance, as it accounted for about half of female and more than half of male heterosexual infections in 2006–2014. [17] Instead of a general assumption that all HIV infections had occurred as a result of transmission within a city or country, we have focussed on modelling the dual occurrence of local and non-local infections. With a highly mobile population, the heterosexual HIV epidemic could be under strong influences of non-locally acquired infections.

Our modelling results showed that even in the absence of new interventions, the annual number of new diagnoses can be halved if there’s zero non-local infections, the results echoing other modelling studies which have incorporated mobility as a parameter. [26–28] Our results further highlighted the differential influence of gender, suggesting that HIV-infected male was a major driver of the HIV epidemic in female, but not vice versa. In our simulation, the effect of high risk sex partnership on the epidemic growth was moderate, compared to results in network-based studies published elsewhere. [12, 29] To control on-going heterosexual transmissions, new interventions targeting male appears to be crucial.

As ART could reduce heterosexual HIV transmission, [20] treatment-based interventions are potentially useful. The importance of linkage to care and retention in care has been highlighted in a study in South Africa [25] and the United States [30]. Our modelling results suggested that enhancing retention in care to 100% or launching HIV testing campaigns with immediate treatment could reduce new infections by 5–7% in male and 9–10% among female by 2023. We simulated the impact of setting up periodic HIV testing campaign (at 5-year intervals), which is theoretically less costly than an annual campaign. If a 100% diagnosis rate is achieved, very low epidemic growth could result. The benefit would be even bigger if periodic testing is combined with immediate treatment. Whereas epidemic control have been concluded in other studies, [23, 31] a test-and-treat strategy in Hong Kong is not anticipated to achieve such significant outcome. The discrepancy might be due to the current high treatment coverage and high proportion of SVL in Hong Kong, leaving limited rooms of improvement. [18] In addition, a high proportion of non-locally acquired infection among the heterosexual male could attenuate the impact of any forms of test-and-treat interventions.

We acknowledge that there’re limitations in our study. The compartmental design might have underestimated the impact of concurrency. Nonetheless, our projected curves were similar to the epidemic curves derived from reported numbers. On the contrary, network structured models might overestimate the impact of concurrency by ignoring the potentially significant reduction of transmission risk after diagnosis and treatment initiation. [20, 32] Other limitations of our study subsuming bisexual population to MSM. Bisexual individuals accounted for only 4% of diagnosed HIV cases, [17] and less than 5% of MSM [unpublished data]. In places like neighbouring Mainland China where some 26% of MSM were bisexual, [33] their inclusion as a separate sub-population would be crucial. We had likewise not considered FSW and their clients separately in the compartment models because of the extremely low (<0.2%, http://www.chp.gov.hk/files/pdf/g220.pdf) HIV prevalence among FSW. Limited by data availability, phylogenetic analysis was only based on the partial polymerase (pol) gene that included protease and reverse transcriptase regions.

As regards parametrization, our study has included non-locally acquired infection as a key parameter. Our approach of not separating overseas infections and those from non-local people in Hong Kong was constrained by data availability. The transmission hazard for high risk sexual partnership was estimated by the formula of random mixing, so as to minimize model uncertainty. This might have underestimated the transmission risk as defined in other studies. [12, 29] We only stratified non-locally acquired individuals by ethnicity, but not for locally acquired individuals, assuming that there was no significant difference of infection risk by ethnicity in local population. This may however not be true, and adjustment is needed in settings with significant difference. Furthermore, model estimation might vary by the different units of hazard estimation. For example, the transmission hazard could be expressed in per-act basis instead of unit of partnership in this study, the results of which are not comparable. The assignment for partnership duration was arbitrary, due to the lack of relevant local data for the definitions. Nonetheless, model estimation on the annual number of new diagnoses and prevalent cases were close to the reported numbers.

In conclusion, enhancing retention in care, launching periodic HIV testing campaign, and offering immediate treatment for all diagnosed persons would be most beneficial for controlling the heterosexual spread of HIV in Hong Kong. Such approach would also be most feasible for introduction within the existing healthcare system. To monitor the epidemic growth in conjunction with the implementation of new interventions, it would be desirable to include indicators reflecting the cascade of care. On the other hand, in view of the potentially high influence of non-locally acquired infections on the heterosexual epidemic, more related information would need to be collected from newly diagnosed cases. To improve our understanding of sexual partnership (concurrent and serial monogamy) and partner exchange rate among heterosexuals, tailored behavioural study regularly conducted locally would be most useful. Finally, while our models were designed for Hong Kong, we believe that the methods and results are applicable for areas with similar socio-ethnic background, HIV epidemic condition and HIV care and surveillance system, such as some Mainland Chinese cities and Taiwan.

## Supporting information

S1 Appendix Description of model parameters, model flow diagram, supplementary tables and supplementary figures. (DOCX)

S1 Table (XLSX)
