# Supplementary material for: The differential impacts of non-locally acquired infections and treatment interventions on heterosexual HIV transmission in Hong Kong
Source: PLoS One. 2020 Aug 13;15(8):e0237433. doi: 10.1371/journal.pone.0237433 (PMC7425942; doi:10.1371/journal.pone.0237433)
Supplement: S1 Appendix — (DOCX) [file pone.0237433.s001.docx]

**The differential impacts of non-locally acquired infections and treatment interventions on heterosexual HIV transmission**

Ngai Sze WONG, Man Po LEE, Ka Hing WONG, Owen T.Y. TSANG, Shui Shan LEE

**S1 Appendix**

**Methods for parameters estimation from clinical dataset**

**Diagnosis rate (vc)**

Assuming all HIV-infected individuals would eventually be diagnosed, reported and recorded in the clinical dataset in Hong Kong, the diagnosis rate can be approximated by the CD4 count at diagnosis. Without treatment, CD4 cells are depleted after infection. The lower the CD4 count, the longer is the interval from HIV infection. We categorized all HIV infected individuals (without AIDS) by CD4 compartments in the model (CD4>500/µL, 351-500/µL, 201-350/µL, ≤200/µL) and counted the total number of individuals at each CD4 category in each year.

We assumed there was a lag of up to 8 years (90% of the interval from seroconversion to AIDS diagnosis) for an individual to get diagnosed. There was 0 lag for those diagnosed with CD4>500/µL, 2 years’ lag for CD4 351-500/µL, 4 years’ lag for CD4 201-350/µL, 6 years’ lag for CD4 ≤200/µL, and 8 years’ lag for AIDS. We then adjusted the total number individuals in each year by the lag time in respective CD4 category.

We estimated the diagnosis rate in three periods, namely 1985-1989 (early epidemic era), 1990-1996 (pre- HAART (highly active antiretroviral therapy) era) and 1997-2004 (HAART era). The diagnosis rate in each period and CD4 compartment was estimated by


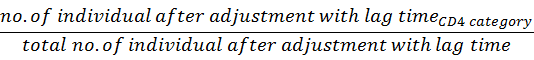


With limited data available, diagnosis rate after 2004 was assumed to be the same as that in the year 2004.

**Pre-treatment disease progression rate (τ)**

The disease progression rate before AIDS was estimated from pre-treatment CD4 measurements in the clinical dataset. CD4 measurements taken after AIDS diagnosis were excluded. CD4 count was categorized as follows: CD4>500/µL, 351-500/µL, 201-350/µL and ≤200/µL. The duration of each HIV-infected individual staying in each CD4 category was calculated as the interval between of 2 measurement dates in the respective category. We took the median duration of all HIV-infected individuals for each CD4 category.

We assumed that HIV-infected individuals progressed to AIDS only when their CD4 dropped below 200/µL. The disease progression rate from CD4≤200/µL to AIDS was estimated from pre-treatment CD4 measurements among individuals with AIDS diagnosis. Duration from CD4≤200/µL to AIDS was defined as the interval between the date of last CD4≤200/µL measurement (before AIDS diagnosis) and the date of first AIDS diagnosis. The median duration was taken as the model parameter.

**Treatment initiation rate (Tx)**

The treatment initiation rate in each year was calculated by dividing the total number of individuals initiated treatment by the total number of individuals without treatment, stratified by CD4 categories.

**Loss to follow-up rate (κ1,** **κ2), viral load suppression (ω) and rebound rate (ψ)**

Individuals were regarded as being lost to follow-up if they did not have CD4 measurement in 3 consecutive years. Before treatment, the rate of loss to follow-up was calculated from dividing the number of patients lost to follow-up by the total number of individuals, stratified by CD4 categories.

After treatment initiation, the status of each individual in each year was defined by a matrix of stage of care (in-care or loss to follow-up) against viral load (suppression or non-suppression). The change of status was marked. The overall annual status changing rate among all individuals was measured by dividing the number of individuals whose specific status has changed in the year by the number of individuals in the respective status in the year.

**Force of infection (λ)**

HIV transmission rate per year in our model was determined by the force of infection (**λ**), which was defined as the rate of heterosexual contacts resulting in transmission(1):

λ =β *φ * I(t) [1]

where **β**= infectious contact rate, **φ**=transmission risk, **I(t)** = number of infected at time t

Reproduction number (**R**) = βφ * N * d [2]

where **N**= total population size, **d**=duration

Combining equation [1] with equation [2], the force of infection in our model can also be:

λ = (/N) * I(t) [3]


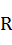

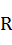

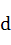

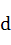


We stratified the reproduction number by the level of risk of sexual partnership and diseases stages for estimating the overall force of infection:

λ = (/N) * I(t) [4]


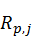

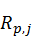

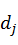

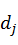


where **p**=level of risk of sexual partnership: the susceptible population was classified into three levels of risk, which are 1) highest risk – concurrent partnership with partners also in concurrent partnership, 2) medium risk – serial monogamy with partners in concurrent partnership, and vice versa, 3) low risk – serial monogamy with partner also in serial monogamy in the respective year. Reproduction number for these levels were estimated with reference to reproduction number formula for random mixing (for highest risk) and serial monogamy (for medium and low risk) in Hollingsworth et al’s study.(2)

And **j**=HIV disease stages (acute infection, chronic infection by CD4 levels, and AIDS) in different stages of HIV care (being undiagnosed, diagnosed, on treatment with or without viral load suppression).(2, 3)

**Supplementary Table 1 Symbol and description for parameters**

| **Symbol** | **Description** | **Value, range** | **Reference** |
| --- | --- | --- | --- |
| **Susceptible individuals** | | | |
| **Net** | Annual net change of adult heterosexuals by gender | It ranges between 16,052 and 41,551 for female, and between 6,285 and 37,853 for male | (4) |
| **N** | Number of adult heterosexuals by gender | Initial number: 1,125,087 for female and 1,630,437 for male | (4) |
| **mm** | Annual mortality rate of adult heterosexuals by gender | male 5.17 per 1000; female 4.3 per 1000 | (4) |
| **CHnonlocal** | Number of non-locally acquired Chinese heterosexuals by gender | It ranges between 0 and 23 for female, and between 0 and 81 for male | (5) |
| **NCHnonlocal** | Number of non-locally acquired non-Chinese heterosexuals by gender | It ranges between 0 and 21 for female, and between 0 and 22 for male | (5) |
| **Infected individuals** | | | |
| **α** | Progression rate from acute infection to chronic infection | 12/6 = 2 | (2) |
| **τ_i_** | Progression rate across CD4 levels in chronic infection (CD4 >500 ⭢ 351-500 ⭢ 201-350 ⭢ ≤200) | Supplementary Table 2 | Clinical data |
| **mu** | Annual mortality rate of AIDS | 0.29483 before 1999, and 0.09483 onward | Clinical data |
| **vc_i_** | Diagnosis rate per year by CD4 levels and AIDS across time (CD4 >500, 351-500, 201-350, ≤200) by gender | Female (range)  CD4 >500: 0.04-0.13  CD4 351-500: 0.04-0.15  CD4 201-350: 0.13-0.38  CD4 ≤200: 0.2-0.42  Male (range)  CD4 >500: 0.03-0.11  CD4 351-500: 0.08-0.15  CD4 201-350: 0.1-0.2  CD4 ≤200: 0.19-0.33  (see S1 table) | Clinical data |
| **dxlf_i_** | Annual rate of pre-treatment loss to follow-up by CD4 levels and AIDS across time | Range:  CD4 >500: 0-1  CD4 351-500: 0-1  CD4 201-350: 0-1  CD4 ≤200: 0-1  AIDS: 0-0.44  (see S1 table) | Clinical data |
| **bc_i_** | Annual rate of pre-treatment back to care by CD4 levels and AIDS across time | Range:  CD4 >500: 0-0.02  CD4 351-500: 0-0.04  CD4 201-350: 0-0.08  CD4 ≤200: 0.04  AIDS: 0-0.02  (see S1 table) | Clinical data |
| **Tx_i_** | Annual rate of treatment initiation by CD4 levels and AIDS across time | Range:  CD4 >500: 0-0.31  CD4 351-500: 0-0.28  CD4 201-350: 0-0.63  CD4 ≤200: 0-0.86  AIDS: 0-0.91  (see S1 table) | Clinical data |
| **ω** | Annual rate of changing status from NSVL to SVL | It ranges between 0 and 0.7  (see S1 table) | Clinical data |
| **ψ** | Annual rate of rate of changing status from SVL to NSVL | It ranges between 0 and 0.75  (see S1 table) | Clinical data |
| **γ** | Annual rate of rate of changing status from lost to follow-up to NSVL | It ranges between 0 and 0.6  (see S1 table) | Clinical data |
| **κ1** | Rate of rate of changing status from NSVL to lost to follow-up | It ranges between 0 and 0.08  (see S1 table) | Clinical data |
| **κ2** | Rate of rate of changing status from SVL to lost to follow-up | It ranges between 0 and 0.04  (see S1 table) | Clinical data |
| **Population mixing#** | | | |
| **Cm** | Duration of partnership at medium risk for male | 6.31 years [fitted] | Varied between 0.2 and 10 years in model calibration. |
| **Cf** | Duration of partnership at medium risk for female | 4.21 years [fitted] | A factor of **Cm** which varied between 0.5 and 1.5 in model calibration. |
| **Stabled** | Duration of partnership at low risk for both male and female | 30 years [fitted] | Varied between 5 and 30 years in model calibration. |
| **Pcc** | Proportion of partnership at high risk in heterosexual population (30% of male and 20% of female had concurrent sex partner(s) and form partnership) | 6% | (6) |
| **Pss** | Proportion of partnership at low risk in heterosexual population | 40% [fitted] | Varied between 40% and 80% in model calibration |
| **Psc** | Proportion of partnership at medium risk in heterosexual population (1 - **Pcc** – **Pss**) | 54% |  |

#**Partnership at high risk**: both male and female had concurrent sex partner(s) and form partnership, and assumed the formation of partnership was in random mixing

**Partnership at low risk**: both male and female have maintained stable monogamous relationship with their spouse

**Partnership at medium risk**: either one had concurrent sex partner(s)

**Supplementary Table 2 Transmission hazard and duration by disease stages and cascade of HIV care**

| **Stage** | **Transmission hazard*** | **Duration** |
| --- | --- | --- |
| **Undiagnosed** |  |  |
| Acute infection | 2.76 (2) | 0.5 years (2) |
| Chronic infection | 0.106 (2) | 6.5 years, including  22 months for CD4>500/μL,  21 months for CD4 351-500/μL,  19 months for CD4 201-350/μL,  16 months for CD4 ≤200/μL  [clinical data] |
| AIDS | 0.76 (2) | 0.75 years (2) |
| **Diagnosed** |  |  |
| Chronic infection and AIDS | 0.036 (7) | rate per year |
| Initiated treatment with NSVL | 0.0179 (3) | rate per year |
| Initiated treatment with SVL | 0.001253^a^ | rate per year |
| Lost to follow-up | 0.106^b^ | rate per year |

*unit: per person per year;

^a^ Assumed 93% reduction of transmission risk with NSVL (3, 8)

^b^ Assumed to be the same as undiagnosed chronic infection

NSVL: non-suppressed viral load (>500/mL); SVL: suppressed viral load (≤500/mL)

**Supplementary Figure 1. Compartmental model diagram**

**
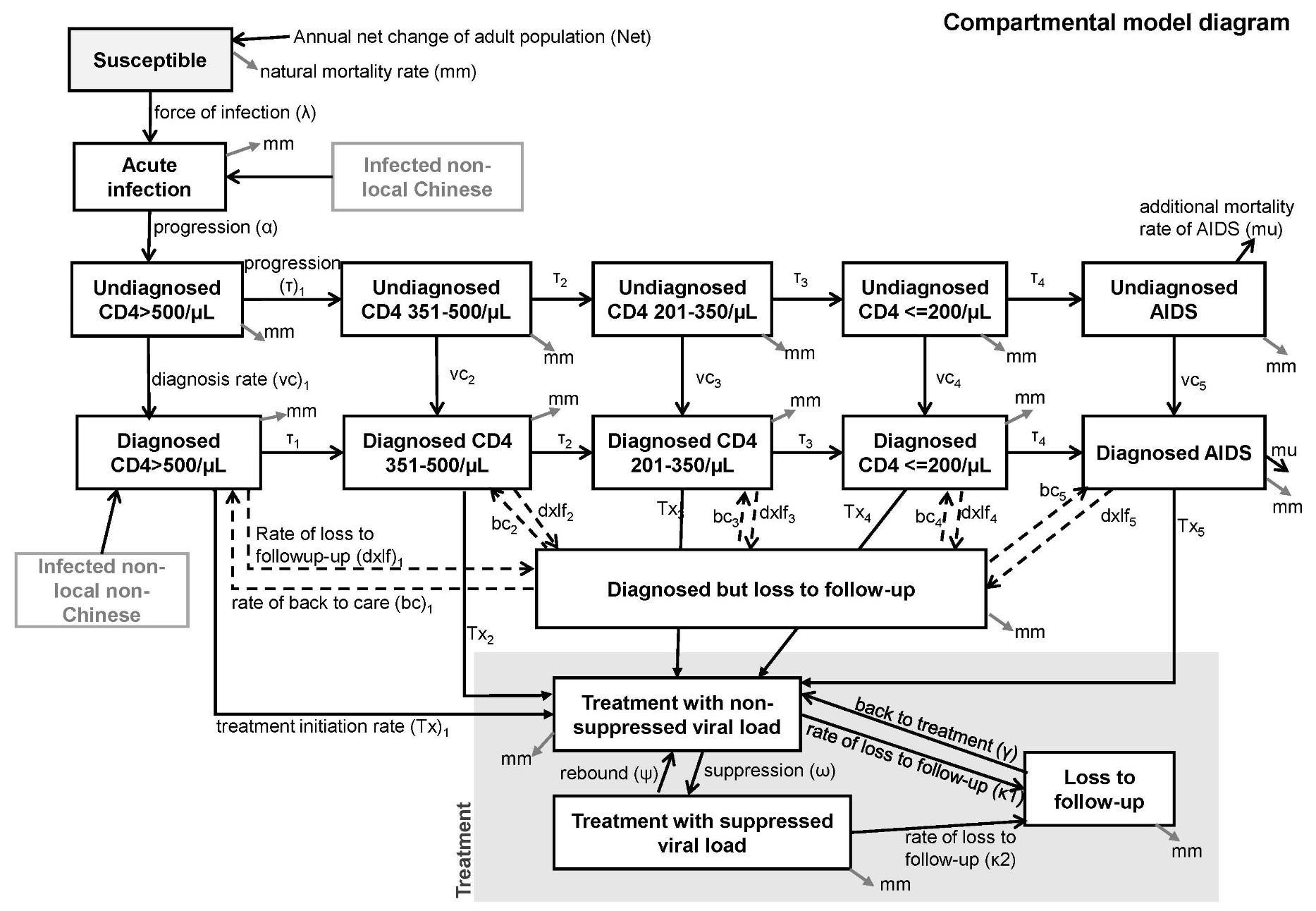
**

**Supplementary Figure 2. HIV sequences clusters drawn as social networks, coloured by the mode of transmission**

**
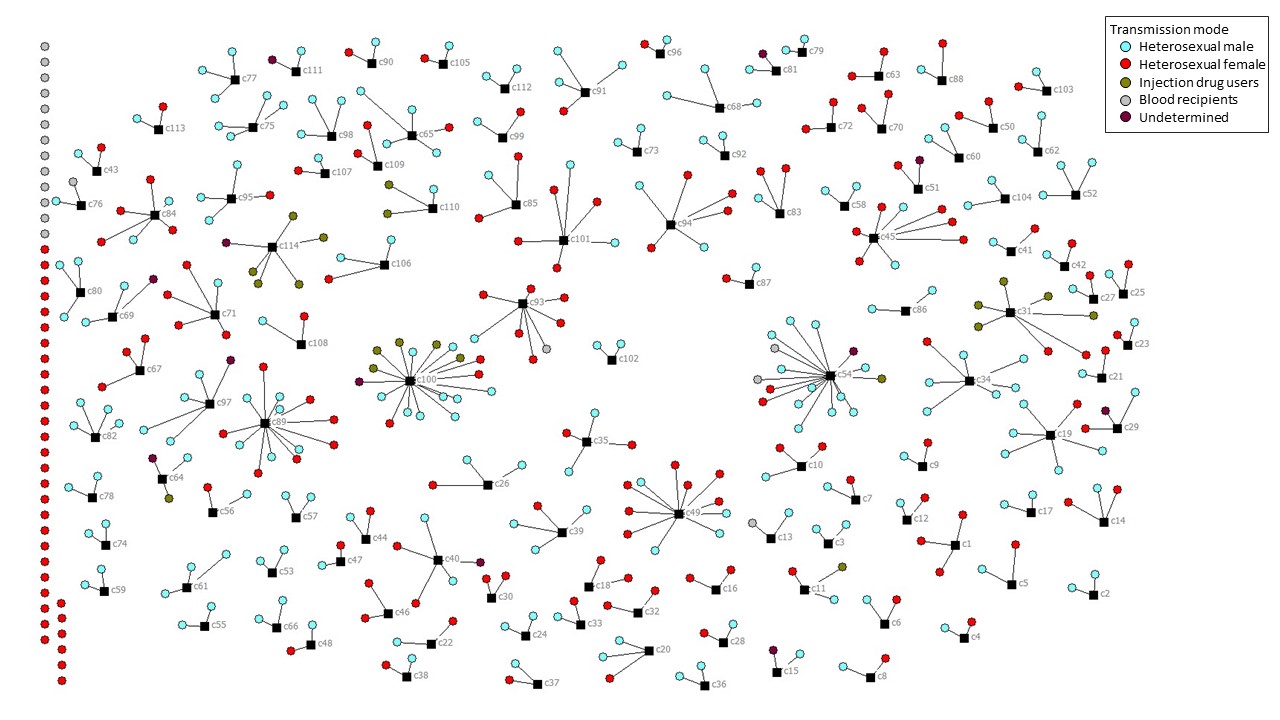
**

**Supplementary Figure 3. Sensitivity analyses with varying the proportion of non-locally acquired infections above base-case from 2012**

**
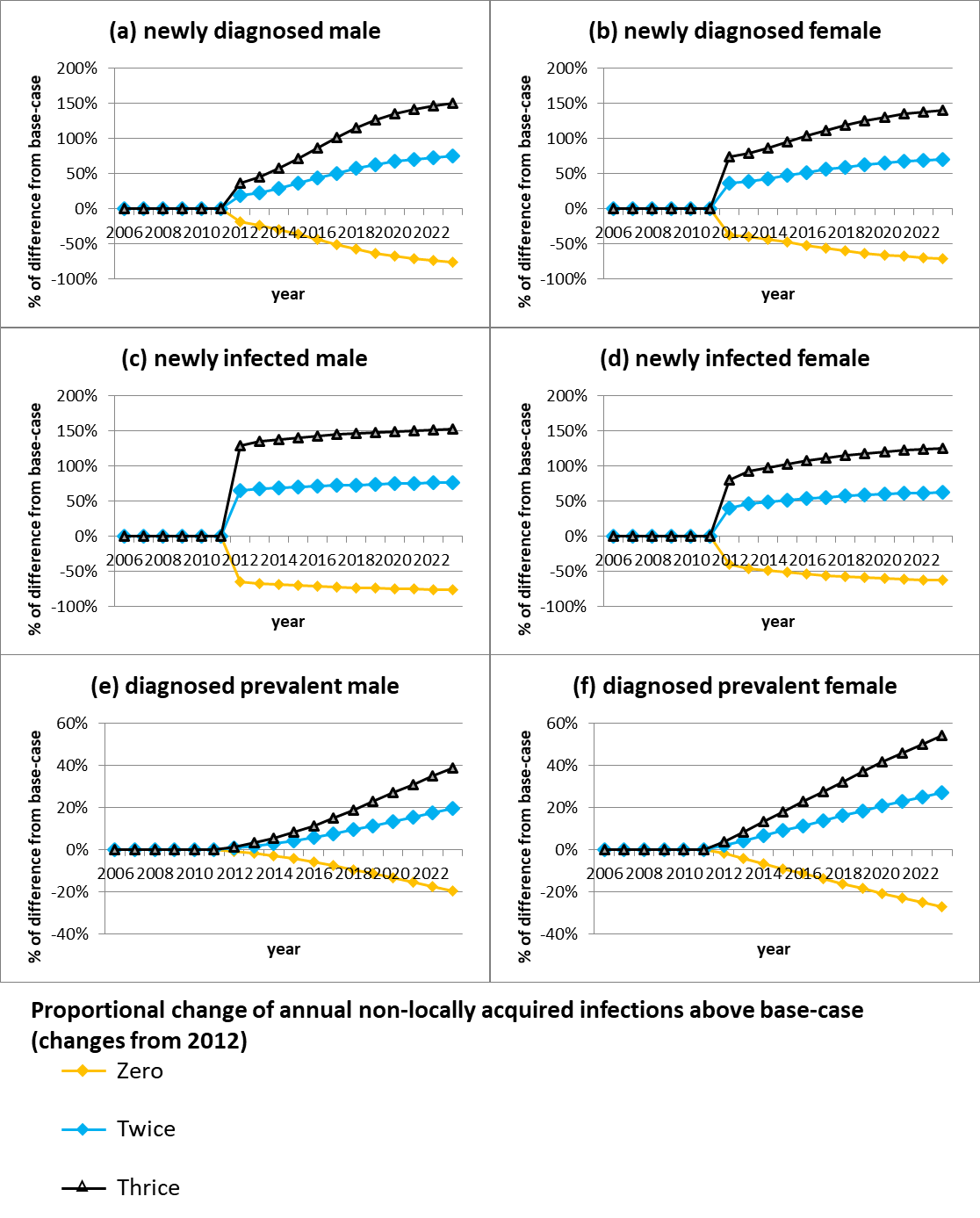
**

**Supplementary Figure 4. Sensitivity analyses with varying the combinations of sexual partnership by gender above base-case from 1983**

**
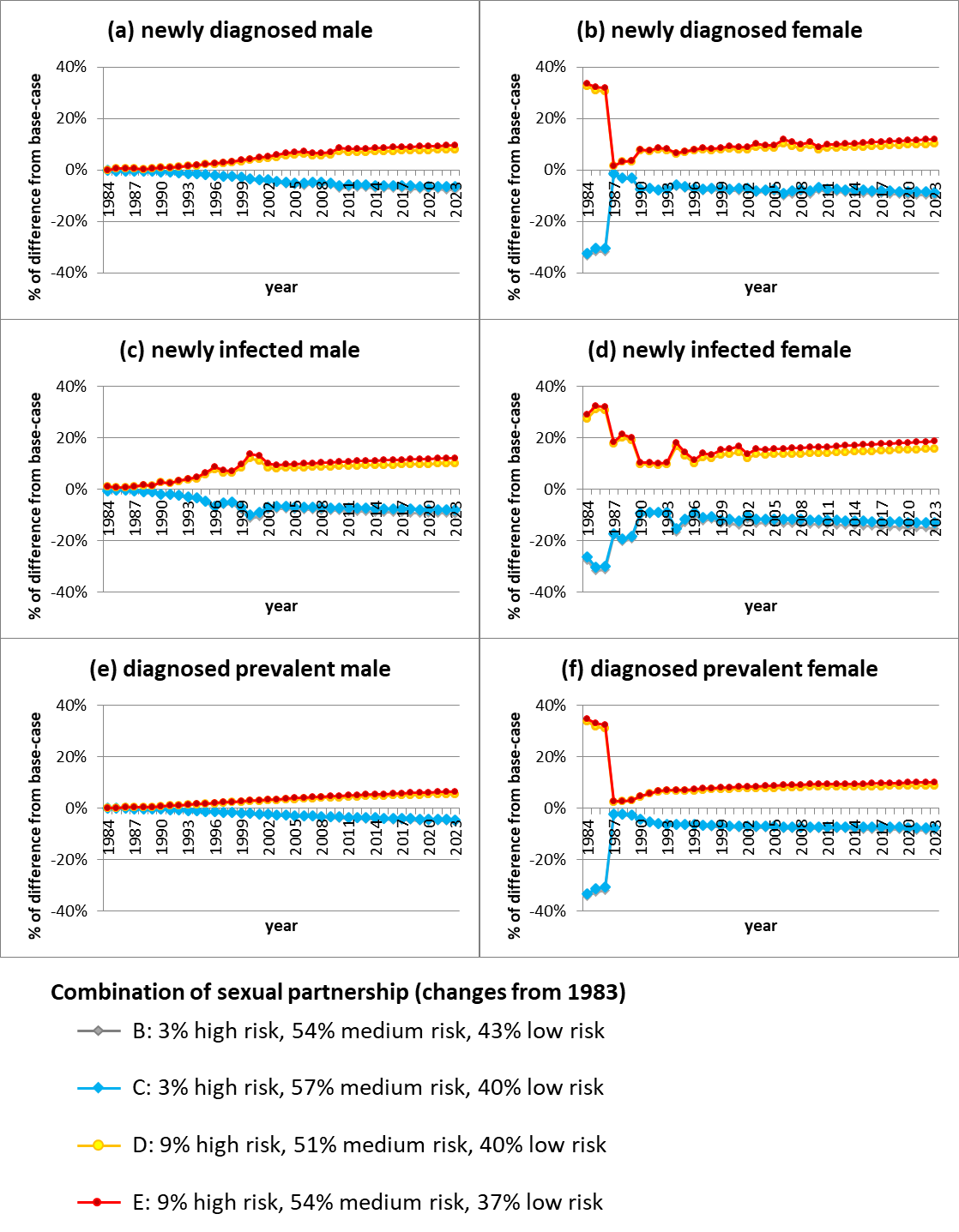
**

**Supplementary Figure 5. Sensitivity analyses with varying the combinations of sexual partnership by gender above base-case from 2012**

**
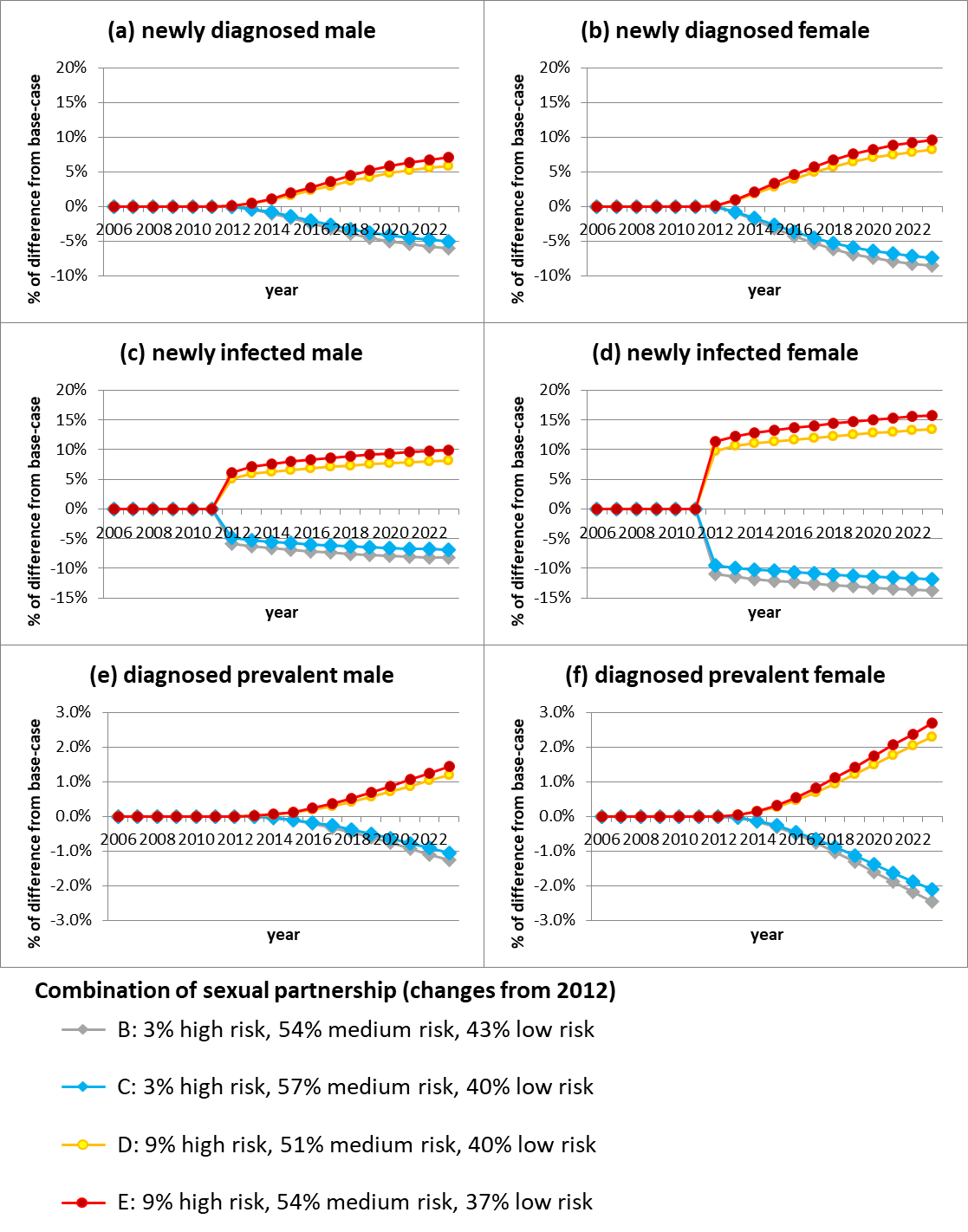
**

**Supplementary Figure 6. Sensitivity analyses with varying the proportion of non-locally acquired infections by gender above base-case from 1983**

**
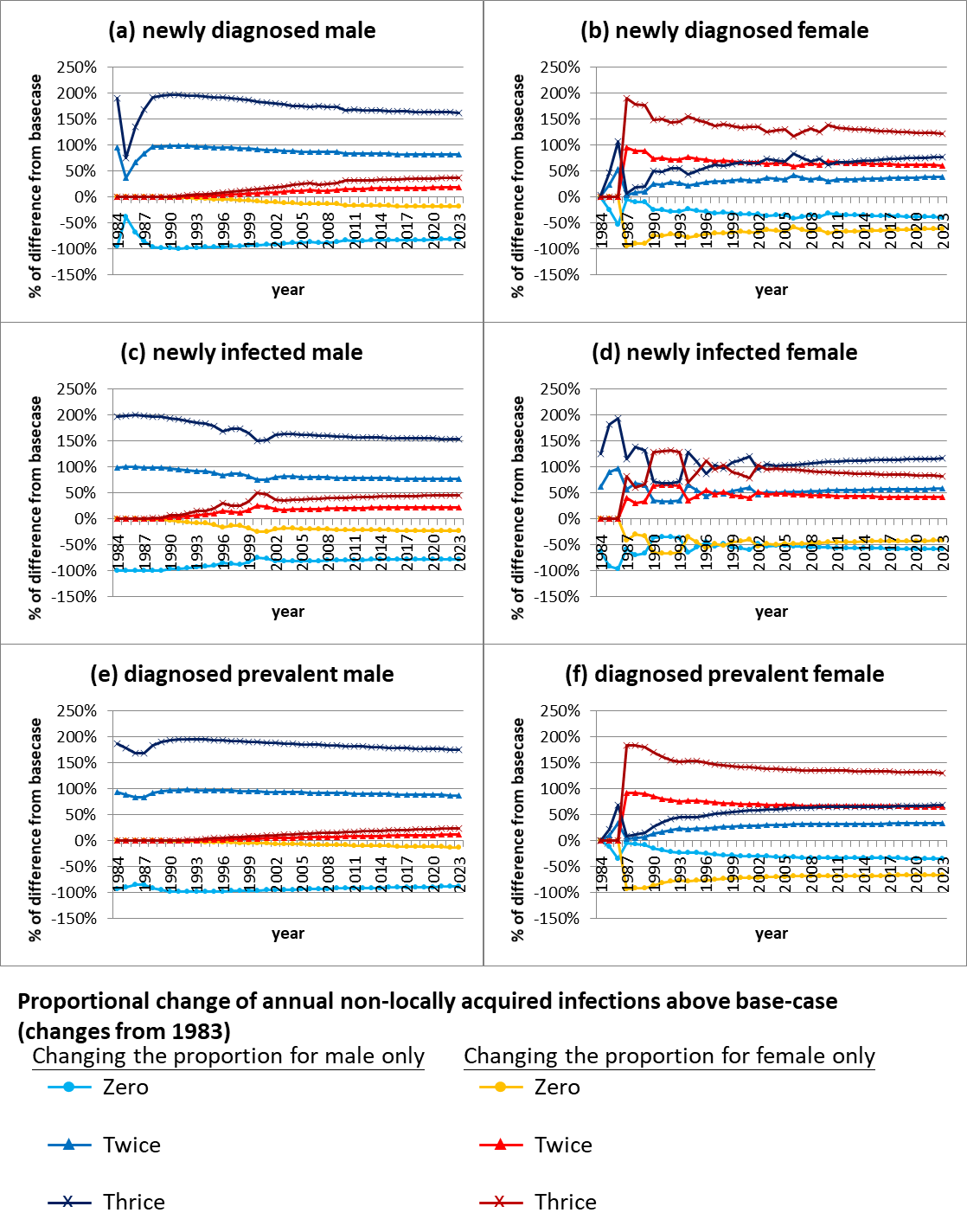
**

**Reference**

1. Vynnycky E, White R. An introduction to infectious disease modelling: OUP Oxford; 2010.

2. Hollingsworth TD, Anderson RM, Fraser C. HIV-1 transmission, by stage of infection. The Journal of Infectious Diseases. 2008 Sep 1, 2008;198:687-93.

3. Jean K, Gabillard D, Moh R, Danel C, Fassassi R, Desgrées-du-Loû A, et al. Effect of early antiretroviral therapy on sexual behaviors and HIV-1 transmission risk among adults with diverse heterosexual partnership statuses in Côte d'Ivoire. The Journal of Infectious Diseases. 2014 Feb 1, 2014;209:431-40.

4. Census and Statistics Department HKSAR. Demographic trends in Hong Kong 1981–2011. 2012 [cited 2015; Available from: http://www.censtatd.gov.hk/hkstat/sub/sp150.jsp?productCode=B1120017

5. Department of Health HKSAR. HIV surveillance report – 2013 update. Hong Kong December 2014.

6. Chang HC, Ruan FF, Chien SC. The study in the survey of the marriage and the affairs cognition: for married and single men and women. 樹德人文社會電子學報第九卷第一期民國一百零二年三月

7. Baggaley RF, White RG, Hollingsworth TD, Boily MC. Heterosexual HIV-1 infectiousness and antiretroviral use: systematic review of prospective studies of discordant couples. Epidemiology. 2013 Jan;24(1):110-21.

8. Cohen MS, Chen Y, McCauley M, Gamble T, Hosseinipour MC, Kumarasamy N, et al. Final results of the HPTN 052 randomized controlled trial: antiretroviral therapy prevents HIV transmission. IAS 2015 8th Conference on HIV Pathogenesis, Treatment and Prevention. Vancouver, Canada; 2015. p. 9.
